# Cytomegalovirus-Induced Colitis Presenting as Acute Appendicitis in an Immunocompetent Patient With Ulcerative Colitis

**DOI:** 10.14309/crj.0000000000001682

**Published:** 2025-05-02

**Authors:** Alessandro Pedicelli, Amine Zoughlami, Olga Aleynikova, Christina Greenaway, Corey Miller

**Affiliations:** 1 Department of Medicine, Division of Gastroenterology and Hepatology, Jewish General Hospital, McGill University, Montreal, Québec, Canada; 2 Department of Pathology, McGill University, Montreal, Québec, Canada; 3 Department of Medicine, Division of Infectious Diseases, Jewish General Hospital, McGill University, Montreal, Québec, Canada

**Keywords:** cytomegalovirus, inflammatory bowel disease, ulcerative colitis, appendicitis

## Abstract

We report a rare case of cytomegalovirus (CMV) colitis presenting radiologically as acute appendicitis in an immunocompetent 71-year-old man known for ulcerative colitis. Although initial imaging revealed acute appendicitis, a lack of clinical improvement with antibiotic therapy prompted further evaluation with colonoscopy, which led to the diagnosis of CMV colitis on a background of mild ulcerative colitis flare. Most individuals infected by CMV will exhibit lifelong latency; however, the virus can reactivate under impaired immune status (eg, transplant recipients, those receiving immunosuppressive therapy). Patients with underlying inflammatory bowel disease are especially at risk of CMV colitis during an acute flare. This case illustrates a unique presentation of CMV colitis in an immunocompetent host, with initial imaging studies revealing acute appendicitis.

## INTRODUCTION

Cytomegalovirus (CMV) is a double-stranded DNA virus with a global seroprevalence of 45%–100%.^1^ Most individuals infected by CMV will exhibit lifelong latency; however, the virus can reactivate under an impaired immune state, most commonly in transplant recipients, those receiving immunosuppressive therapy, or patients with immunosuppressive diseases such as human immunodeficiency virus (HIV)/AIDS. Reactivation of the virus can lead to widespread end organ damage including hepatitis, colitis, pneumonitis, myocarditis, and encephalitis.^2^ Despite the high seroprevalence of CMV, clinically significant CMV disease rarely occurs in immunocompetent patients.^2^ However, CMV colitis is more likely to occur in immunocompetent individuals with inflammatory bowel disease (IBD), diabetes mellitus, and chronic kidney disease.^3^ We report a case of CMV colitis presenting as acute appendicitis in an immunocompetent patient known for ulcerative colitis (UC).

## CASE REPORT

A 71-year-old man presented to the emergency room with a history of 10 days of intermittent fevers (objectified fever of 40°C at home), lower abdominal discomfort, and constipation of 4 weeks duration. The patient was known for UC, type 2 diabetes mellitus, dyslipidemia, and benign prostatic hyperplasia. Medications included oral and topical mesalamine, metformin, sitagliptin, gliclazide, canagliflozin, aspirin, rosuvastatin, and alfuzosin. He had no allergies and did not smoke or drink alcohol.

The patient's vital signs revealed a fever of 38.2°C with all other parameters within normal limits. He was alert and oriented. His abdomen was soft, with some right lower quadrant fullness but no significant tenderness. The remainder of his physical examination was noncontributory.

Initial biochemical workup revealed anemia (hemoglobin 125 g/L), hypoalbuminemia (31 g/L), elevated alanine transaminase (102 IU/L), and elevated C-reactive protein (45 mg/L) (see Table 1 for further details). Computed tomography scan of the abdomen and pelvis revealed a retrocolic appendix with wall thickening, increased diameter, surrounding fat stranding, and edema of the cecal base, consistent with acute uncomplicated appendicitis. In addition, there was evidence of rectal wall edema, in keeping with possible proctitis. The patient was subsequently admitted by the general surgery service and was started on broad-spectrum antibiotics (piperacillin–tazobactam) and intravenous fluids for an acute appendicitis.

**Table 1.** Pertinent laboratory values at presentation and follow-up

Lab test	At presentation	Follow-up	Reference range
Hemoglobin (g/L)	125	131	140–175
White blood cells (×10^9^/L)	6.5	7.4	4–11
Neutrophils (×10^9^/L)	4.2	5.3	1.8–7.5
Lymphocytes (×10^9^/L)	1.8	1.7	1.2–3.5
Monocytes (×10^9^/L)	0.4	0.2	0.2–0.8
Eosinophils (×10^9^/L)	0.0	0.1	0.0–0.5
Basophils (×10^9^/L)	0.1	0.1	0.0–0.2
Platelets (×10^9^/L)	197	268	150–400
CRP (mg/L)	45	0.6	0–10
ALT (IU/L)	102	53	5–40
Albumin (g/L)	31	43	35–51
ALP (IU/L)	73	60	40–125
Total bilirubin (mmol/L)	7	6	3–17
CMV quantitative PCR copies/mL	64,822	26	<20 copies/mL

ALP, alkaline phosphatase; ALT, alanine aminotransferase; CMV, cytomegalovirus; CRP, C-reactive protein; PCR, polymerase chain reaction.

Over the next 24 hours and despite antibiotic therapy, the patient remained intermittently febrile and began experiencing diarrhea with mild hematochezia. His C-reactive protein value continued to rise (maximally 65 mg/L). An initial infectious workup was negative for hepatitis A, B, and C, Epstein–Barr virus, HIV, syphilis, as well as for bacterial bloodstream and urine infections. CMV serum IgG, IgM, and viral load were requested.

**Figure 1.**
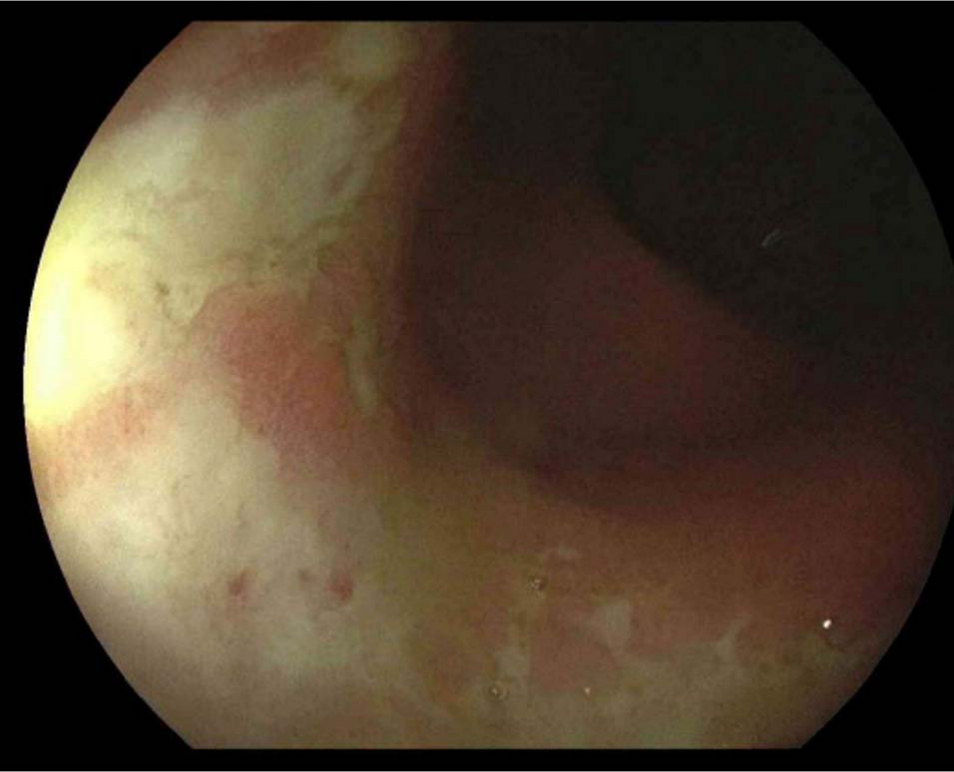
Ulcerated and inflamed distal colon.

**Figure 2.**
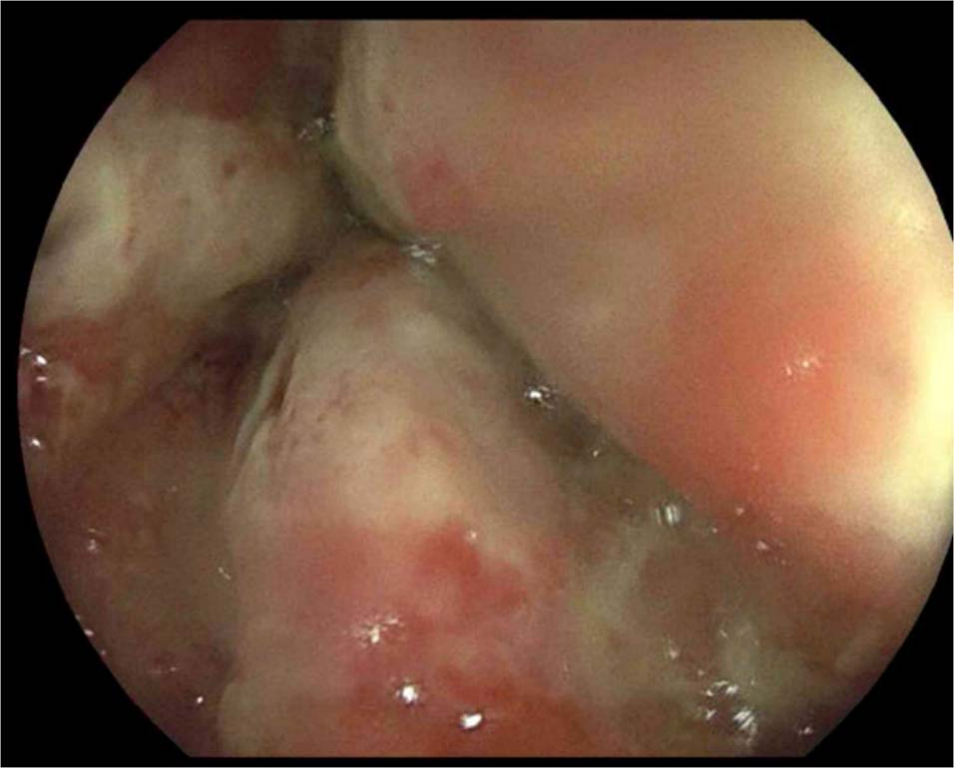
Ulcerated and inflamed distal colon.

**Figure 3.**
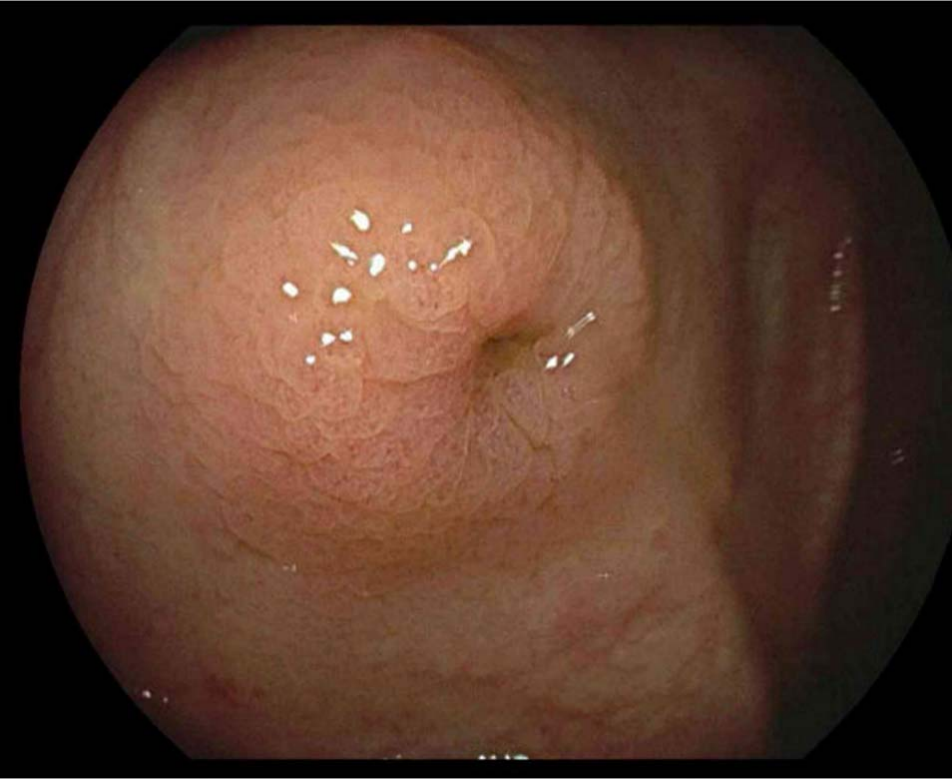
Appendiceal orifice with surrounding edema.

Given the new onset of diarrhea and hematochezia, a colonoscopy was performed on day 3 of his admission. It revealed severely ulcerated proctitis in the distal 8 cm of the colon (Mayo UC endoscopic score = 3), scattered erosions throughout the remaining colonic segments, and edema surrounding the appendiceal orifice (see Figures 1–3). A diagnosis of acute IBD flare and incidental acute appendicitis was entertained, but the decision to initiate immunosuppressive therapy was delayed until pathologic examination of the biopsies taken during colonoscopy was completed.

The next day, serologies resulted positive for CMV IgG and IgM. Given the atypical course with ongoing fever for 21 days despite antibiotics, mild transaminitis and positive CMV IgM and IgG, intravenous ganciclovir therapy was started for suspected CMV colitis on a background of mild-to-moderate UC flare. Piperacillin–tazobactam was discontinued.

**Figure 4.**
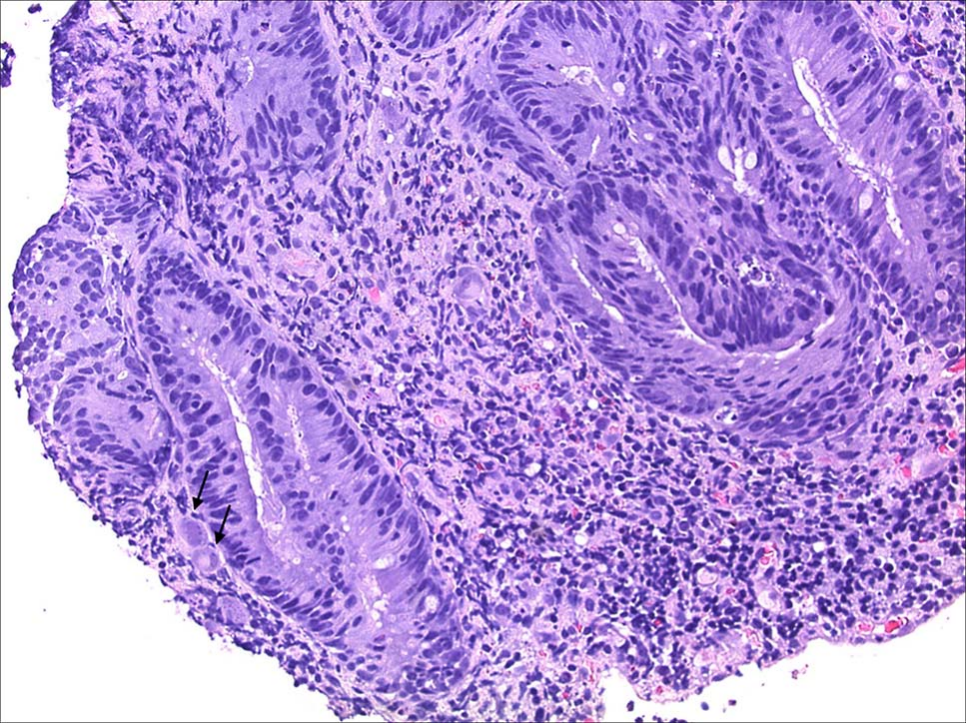
Colonic mucosa with marked cells showing characteristic features of cytomegalovirus infection, including cytomegaly, enlarged nuclei, and typical “owl-eye” intranuclear inclusions (200× magnification).

**Figure 5.**
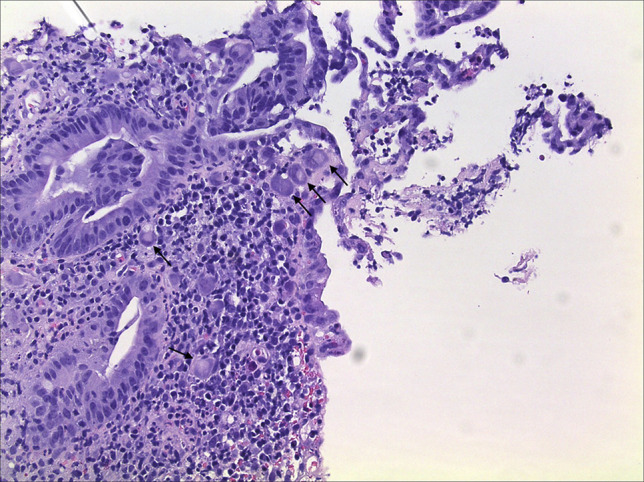
Colonic mucosa with marked cells showing characteristic features of cytomegalovirus infection, including cytomegaly, enlarged nuclei, and typical “owl-eye” intranuclear inclusions (200× magnification).

**Figure 6.**
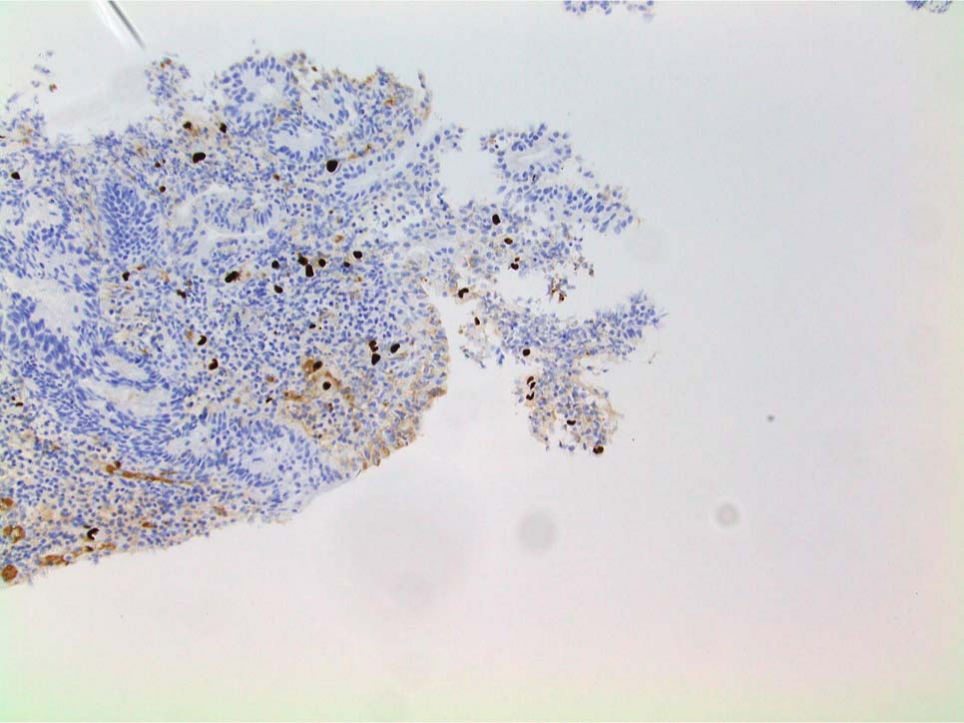
Confirmatory immunohistochemistry staining done with cytomegalovirus antibody (200× magnification).

Within 48 hours of receiving ganciclovir, the fever resolved, and the diarrhea improved. The examination of colonic biopsies revealed focal active colitis in the ascending, transverse, and descending colon with positive CMV immunostaining (see Figures 4–6). In addition, there was a background of mild-to-moderate chronic active colitis in the sigmoid colon and rectum, indicating UC disease activity. Biopsies of the appendiceal base revealed only congested colonic mucosa. Subsequently, results of the quantitative serum CMV viral load showed 64,822 copies/mL (normal <20 copies/mL).

Over the ensuing days, the patient's diarrhea and hematochezia resolved, and he was discharged home on oral valganciclovir and his home regimen of oral and topical mesalamine. At follow-up 2 weeks after discharge, the patient continued to have no gastrointestinal symptoms, his C-reactive protein and alanine aminotransferase had normalized, and quantitative CMV viral load had decreased to 26 copies/mL.

## DISCUSSION

We report a rare case of CMV colitis presenting as acute appendicitis in context of a UC flare in an immunocompetent host. The case is remarkable for a few reasons.

First, the patient was immunocompetent at the baseline, having a normal immune system and no exposure to systemic corticosteroids or immunomodulating medications before presentation. Although clinically dormant in most immunocompetent hosts, CMV reactivation has long been associated with UC, usually occurring in context of severe flares requiring systemic immunosuppression or colectomy.^4^ The prevalence of reactivation seems to mirror flare severity, with more than 30% of steroid-refractory patients exhibiting evidence of concurrent colonic CMV disease.^5^ This case is atypical in that the underlying UC flare was mild-to-moderate in nature, and the patient exhibited clinical CMV colonic disease even in the absence of steroid and/or immunomodulator therapy for IBD.

Furthermore, the patient's CMV colitis presented uncharacteristically with clinical and radiologic findings of acute appendicitis, and he only developed the more typical symptoms of diarrhea and hematochezia later in his admission. A recent review of CMV disease of the gastrointestinal tract^1^ reports that immunocompetent patients can present with features not typical of gastrointestinal infections, including ischemic enterocolitis and vasculitis-like symptoms. Most CMV appendicitis cases occur in patients with underlying immune system dysfunction, including HIV/AIDS, solid-organ and hematopoietic stem cell transplant recipients.^6–8^ There are only 4 other published cases of CMV appendicitis occurring in immunocompetent hosts, thus adding to the distinctiveness of this case.^9^ The presentation and disease course of the other cases in the literature resemble our own in that the immunocompetent patients presented with atypical gastrointestinal symptoms, had radiologic evidence of acute appendicitis, did not improve with standard antibiotic therapy, and were subsequently diagnosed with primary CMV infection of the appendix and/or colon, which then improved with adequate antiviral therapy.^9^

Whether CMV reactivation is a consequence of an acute UC flare or plays an active role in propagating the underlying IBD remains to be fully elucidated. Still, there is a growing body of evidence that treatment of underlying CMV colitis, when discovered during an acute UC flare, is indicated and can improve outcomes in the form of lower colectomy rates, especially for patients with high CMV tissue viral loads.^10^ As such, gastroenterologists must maintain a high degree of suspicion for comorbid CMV colitis when assessing their patients with UC presenting with acute IBD symptoms, as early recognition and treatment can importantly impact clinical outcomes.

## DISCLOSURES

Author contributions: A. Pedicelli and A. Zoughlami: conceptualization, manuscript writing, editing; O. Aleynikova: editing, pathology slides; C. Miller, C. Greenaway: conceptualization, editing. A. Pedicelli is the article guarantor.

Financial disclosure: None to report.

Informed consent was obtained for this case report.
